# ProteoBoostR: an interactive framework for supervised machine learning in clinical proteomics

**DOI:** 10.1186/s12014-026-09582-8

**Published:** 2026-01-24

**Authors:** Annika Topitsch, Niko Pinter, Tilman Werner, Katja Nelson, Tobias Fretwurst, Oliver Schilling

**Affiliations:** 1 Institute for Surgical Pathology, Medical Center, Medical Faculty, University of Freiburg, University of Freiburg, 79106 Freiburg, Germany; 2 Spemann Graduate School of Biology and Medicine (SGBM), University of Freiburg, 79104 Freiburg, Germany; 3 Faculty of Biology, University of Freiburg, 79104 Freiburg, Germany; 4 Department of Oral and Maxillofacial Surgery/Translational Implantology, Medical Center, Medical Faculty, University of Freiburg, University of Freiburg, 79106 Freiburg, Germany; 5 Department of Oral Medicine, Infection, and Immunity, Harvard School of Dental Medicine, Boston, MA 02115 USA; 6 German Cancer Consortium (DKTK) and German Cancer Research Center (DKFZ), 69120 Heidelberg, Germany

**Keywords:** Proteomics, Machine learning, XGBoost, Classification models, Personalized medicine

## Abstract

**Background:**

Mass spectrometry-based proteomics enables high-throughput quantification of thousands of proteins in clinical samples, fueling biomarker discovery for disease diagnosis and prognosis. However, leveraging complex proteomic profiles for predictive modeling often requires advanced machine learning (ML) expertise that many biomedical researchers lack. User-friendly tools are needed to apply state-of-the-art ML algorithms to proteomics data. XGBoost is a powerful tree-based ML algorithm known for high accuracy in classification tasks, and has been successfully used to classify cancer subtypes from multi-omics data.

**Methods:**

We developed ProteoBoostR, a Shiny application that streamlines supervised ML on protein abundance datasets. It allows researchers to train, evaluate and apply XGBoost classification models through an interactive web interface, without requiring coding.

**Results:**

We demonstrate the application of ProteoBoostR for the classification of proteomic subtypes across two independent datasets of glioblastoma multiforme, and for the detection of lung adenocarcinoma in serum. These application examples illustrate how ProteoBoostR can harness proteomic patterns for the stratification of patients.

**Conclusions:**

ProteoBoostR is an open-source application that empowers proteomics researchers to perform advanced ML classification. It can be readily applied to other proteomic datasets and disease contexts, promoting reproducible ML analyses in proteomics and accelerating the translation of omics-based classifiers into clinical research.

**Supplementary Information:**

The online version contains supplementary material available at 10.1186/s12014-026-09582-8.

## Introduction

Mass spectrometry (MS)-based proteomics has become a key technology in biomedical research, offering a direct view of the functional molecular phenotype of cells and tissues [1, 2]. State-of-the-art platforms can quantify thousands of proteins in a single experiment, providing unprecedented opportunities for the discovery of disease biomarkers and therapeutic targets. MS-based proteomics is now widely applied in clinical research for early disease detection, prognosis evaluation, and monitoring of treatment responses [3–5]. Its high throughput, analytical specificity, sensitivity, and depth of coverage make it a preferred approach over targeted assays in many settings. Consequently, large-scale proteomic studies are building molecular profiles across patient cohorts, contributing to precision medicine initiatives [6].

A major challenge, however, lies in interpreting high-dimensional proteomic data to derive clinically actionable insights. While statistical analyses can easily find individual differentially abundant proteins, a complementary approach is to build predictive models that consider the joint patterns of abundance across many proteins. ML methods are well-suited to detect complex, multivariate patterns in omics datasets [7]. In oncology and other fields, ML has been applied to classify disease subtypes, predict patient outcomes, and identify important feature combinations from omics data [8, 9]. In particular, gradient-boosted decision tree algorithms such as XGBoost have gained popularity for their accuracy and efficiency in handling large feature sets [10]. XGBoost has been successfully used to distinguish cancer types and subtypes from multi-omics profiles [11, 12]. Integrating proteomic data with ML has opened a new era in the discovery and validation of cancer biomarkers, enabling researchers to move beyond univariate analysis to robust multi-marker models.

Despite the promise of ML in proteomics, there is a gap between algorithmic advancements and their adoption by life scientists. Deploying an ML model typically requires programming ability, which can be a barrier for experimental researchers. The need for accessible, user-friendly tools is especially important in proteomics, where dataset sizes are large and complex. Interactive web-based platforms have been created in the omics fields, allowing users to perform bioinformatic analyses without coding [13]. In proteomics, several web tools exist for data processing and statistical analysis, but few support advanced machine learning model training with a graphical user interface [14, 15].

To address this, we developed ProteoBoostR, an interactive software tool that makes supervised ML accessible to proteomic and clinical researchers. ProteoBoostR is aimed at bridging the gap between proteomics data and predictive modeling. It allows users to train an XGBoost classification model on their protein abundance matrix to, e.g., distinguish disease subtypes or patient outcomes. The tool is wrapped into a Shiny web application, providing point-and-click access to functionalities that would otherwise require advanced coding expertise.

We demonstrate the potential of ML in proteomics by presenting application examples of ProteoBoostR in the context of glioblastoma multiforme (GBM) and lung adenocarcinoma (LUAD). GBM is an aggressive brain tumor known to be highly heterogeneous and of poor prognosis. Proteomic studies have revealed distinct tumor subtypes and have shown that proteomic signatures can carry biological characteristics beyond genomic classifications. LUAD accounts for the majority of lung carcinomas, which are one of the most prevalent cancers worldwide. While the five-year survival rate is promising in early stages, it markedly decreases in advanced stages, highlighting the significance of early detection. We applied ProteoBoostR to build and evaluate classification models that identify tumor subtypes of GBM and detect LUAD from serum. These examples illustrate the importance of ML in analyzing proteomic data to uncover clinically relevant patterns.

## Methods

**Fig. 1:**
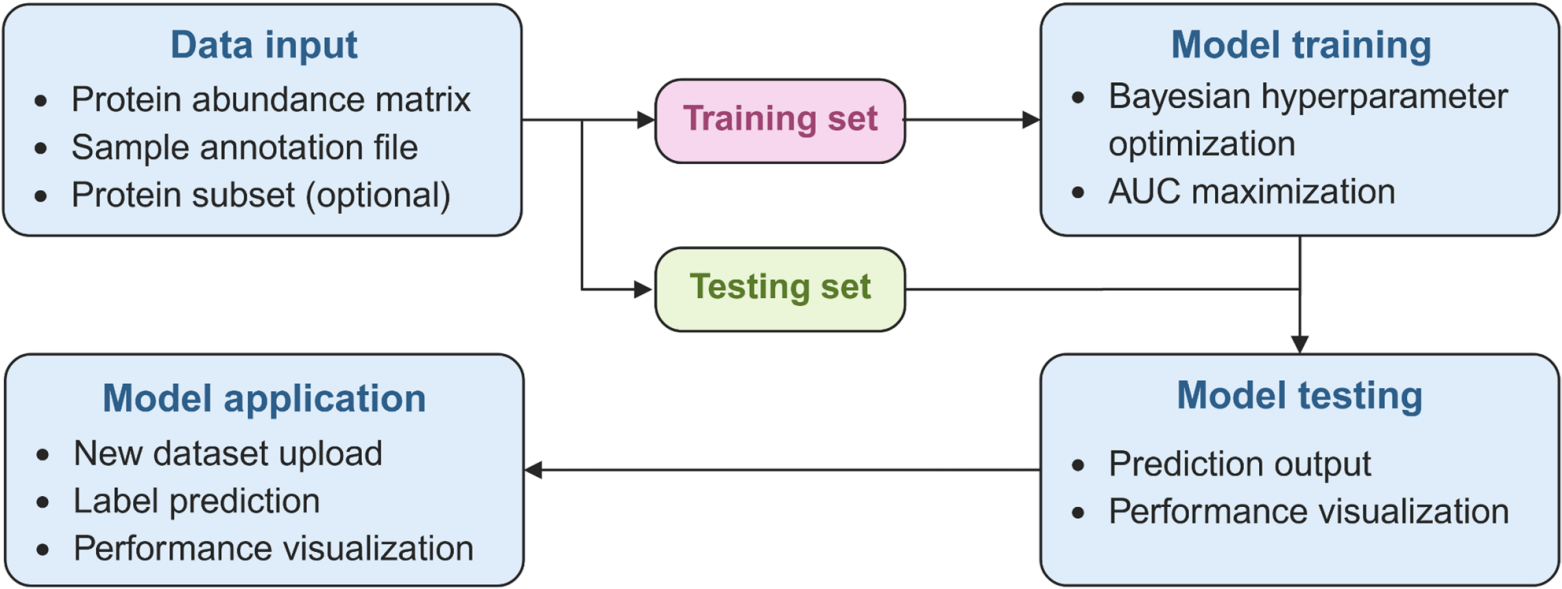
Overview of the ProteoBoostR architecture. The tool uses standard proteomic data inputs, i.e., protein abundance matrix and sample annotation file, and an optional list of a protein subset. After splitting the data in a training and a testing set, Bayesian optimization is employed to tune hyperparameters of an XGBoost model on the training set to maximize cross-validated AUC as the chosen performance metric. With optimized parameters, the XGBoost model is trained on the training set, and its performance is evaluated on the testing set. The tool provides all generated files and figures in an output directory, including the training and testing sets, the predicted class probabilities of the testing set, the confusion matrix of predicted versus true labels, the ROC curve with calculated AUC, and the trained model. The model can then be applied to predict labels in an independent dataset. Predicted class probabilities and labels are reported as well as performance evaluation metrics if true labels are provided

ProteoBoostR is implemented as a Shiny application, combining a client-side user interface with server-side processing of data. The software architecture is configurable, with separate components handling data input, model training, model testing and model application (Fig. 1). For convenience and reproducibility, ProteoBoostR is available as a Docker file that bundles the complete software environment. Users may choose between a Windows or aLinux/MacOS Docker recipe. All required R package dependencies are resolved and installed automatically during the image build. Detailed step-by-step instructions for building and running the container are provided on GitHub (https://github.com/SchillingLabProteomics/ProteoBoostR). Re-execution with this container reproduces all results under the identical software stack.

**Fig. 2:**
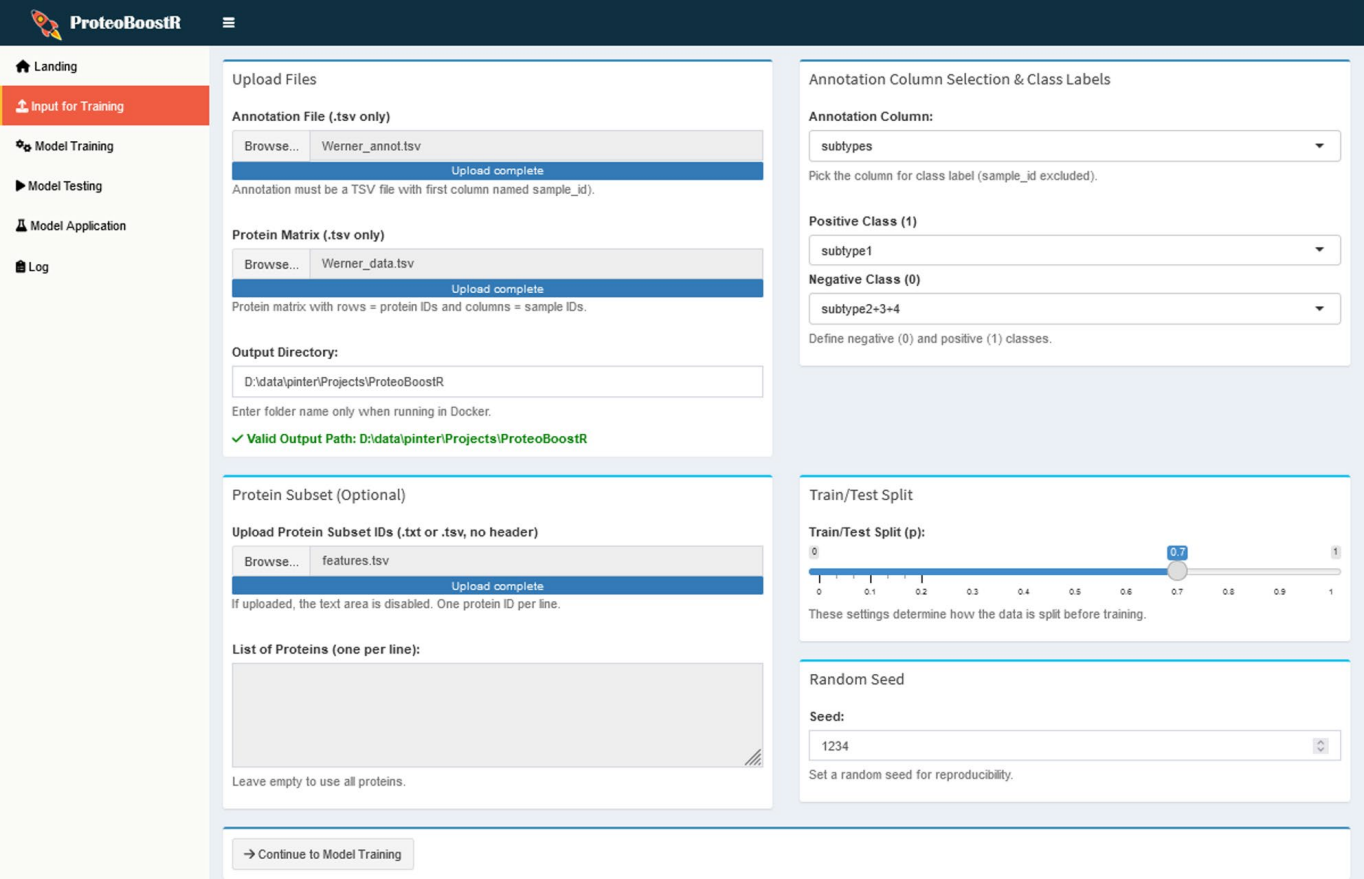
‘Input’ tab. The user uploads sample annotation and protein abundance files, defines an output directory, optionally selects a subset of proteins, and specifies the split ratio, generating the training and testing sets for subsequent model training and testing, respectively

Upon launching ProteoBoostR, the user is presented with a series of tabs guiding them through the pipeline. In the ’Input’ tab (Fig. 2), the user uploads two TSV files: the sample annotation file containing the sample IDs and the known class labels, and the protein abundance matrix with protein IDs in rows and sample IDs in columns. An output directory is specified, where a log file is created immediately and extended throughout the steps. An optional feature is the ability to select a subset of proteins of interest, e.g., if the user wants to train the model on a specific panel of proteins rather than the full proteome. The split ratio of the training and testing set is chosen by percentage. Once the user proceeds to the following tab, the training and testing sets are saved as tsv files within the output directory.

**Fig. 3:**
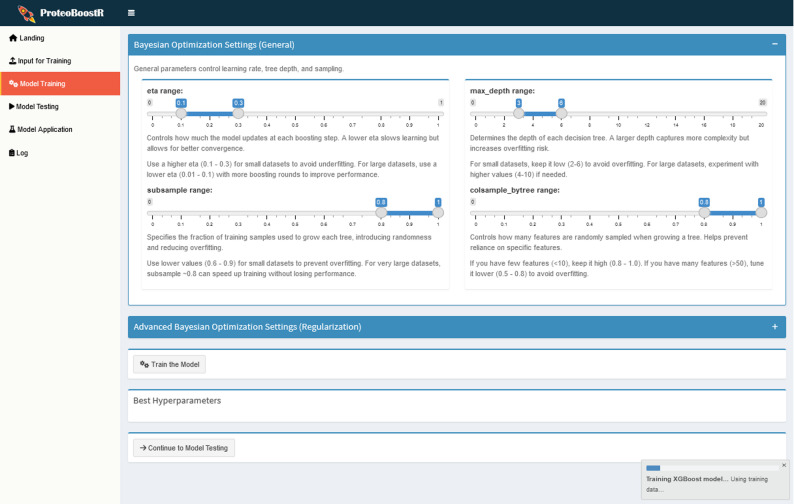
‘Model Training’ tab. The user selects the parameter bounds for Bayesian optimization prior to model training

In the ‘Model Training’ tab (Fig. 3), the user specifies the parameter bounds for Bayesian optimization. Default values and practical recommendations are provided to guide users in adjusting the parameter bounds according to the structure and size of their dataset. Bayesian optimization iteratively tests different hyperparameter combinations and converges on a set that maximizes model performance on the training set through internal cross-validation. The best parameters are used to train the XGBoost model. The used set of parameters and the trained model are saved as tsv and rds files, respectively, within the output directory.

**Fig. 4:**
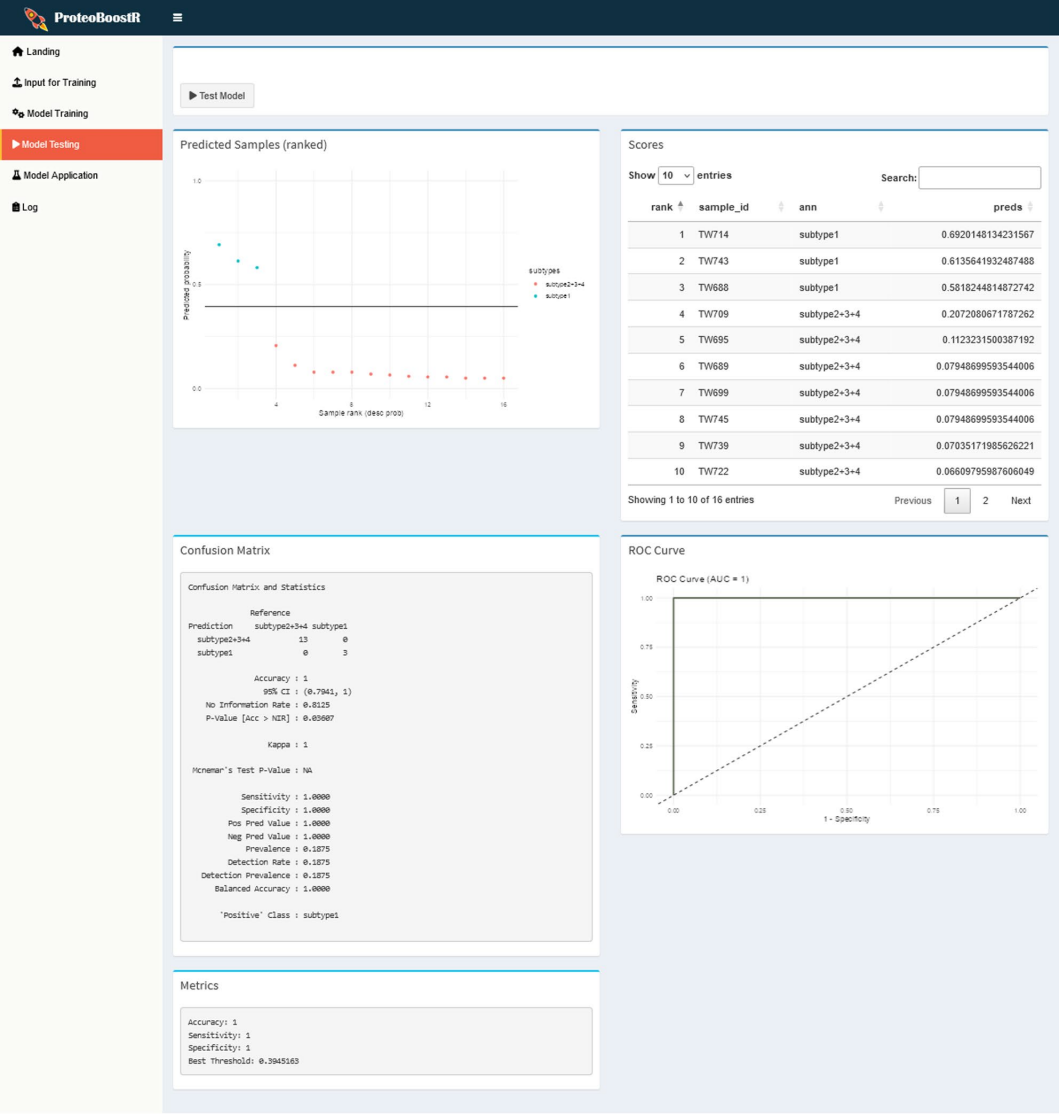
‘Model Testing’ tab. The model is applied to predict labels in the testing set and its performance is evaluated

In the ‘Model Testing’ tab (Fig. 4), the model is evaluated on the held-out testing set. Predicted class probabilities are visualized by rank, a confusion matrix summarizes how many samples were correctly or incorrectly classified into each class, and the performance metrics accuracy, balanced accuracy, sensitivity, and specificity are displayed. The prediction probabilities for the samples in the testing set, the confusion matrix, and the performance metrics are saved as tsv files within the output directory. A ROC curve illustrates the trade-off between sensitivity and specificity across different prediction score thresholds, and the AUC provides a single metric of overall discrimination ability. The plot is saved as a png file within the output directory.

**Fig. 5:**
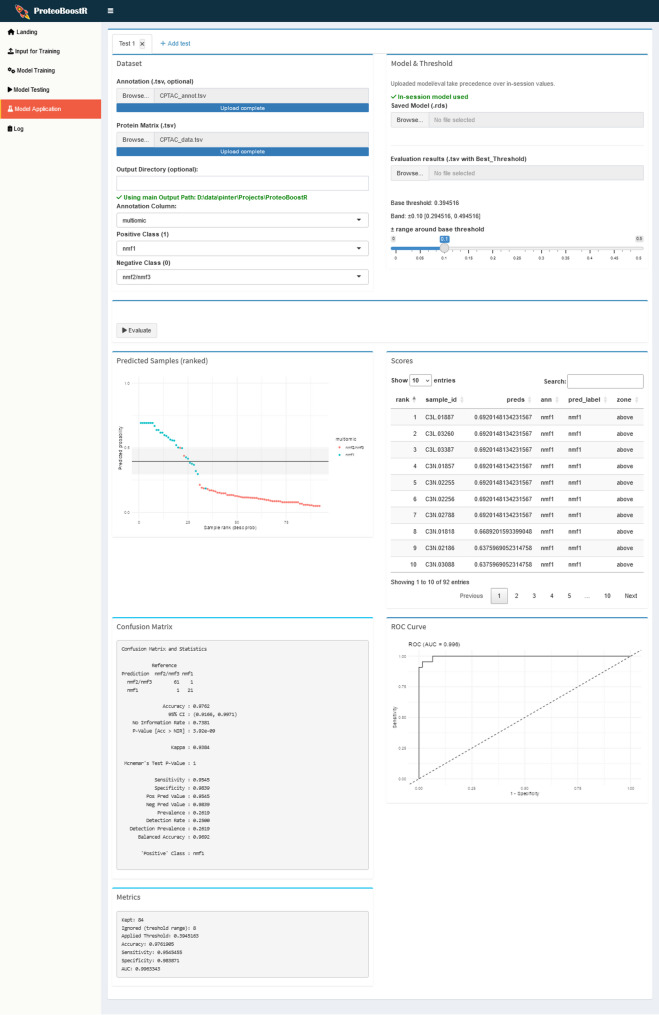
‘Model Application’ tab. The model is applied to predict labels in an independent dataset and its performance is evaluated

In the ‘Model Application’ tab (Fig. 5), an independent dataset is uploaded with or without sample annotations. The model is used to predict labels based on class probabilities. Samples with values close to the classification threshold can be excluded by specifying a range of probabilities to be removed as inconclusive. Predicted class probabilities are reported and visualized by rank. If an annotation file with true labels is provided, the confusion matrix, performance metrics and ROC curve are displayed.

The ‘Log’ tab shows the final output written in the log file as well as the session info containing all used packages and their versions.

## Results

### Application example 1: classification of proteomic subtypes in GBM

We applied ProteoBoostR for the classification of proteomic subtypes. Werner et al. profiled 55 primary GBM tumors and identified four proteomic subtypes using unsupervised hierarchical clustering [16]. The neuronal subtype showed a distinct separation from the other subtypes. We thus aimed to use ProteoBoostR to train a model that could recognize the neuronal subtype from proteomic abundance patterns, effectively condensing the complex proteomic signature into a classifier.

**Fig. 6:**
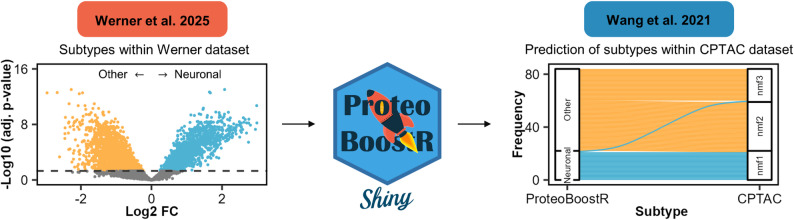
Classification of proteomic subtypes using ProteoBoostR. Volcano plot of the differential abundance analysis between the neuronal and other subtypes in the Werner dataset (left). Predictions of subtypes in the CPTAC dataset match to the reported multi-omic subtypes (right). FC, fold change

We performed a differential abundance analysis to identify changes in protein abundances between the neuronal and the other subtypes (Fig. 6, left). Both the 10 most upregulated and 10 most downregulated proteins in the neuronal subtypes with the highest absolute fold changes were selected as features for model training. ProteoBoostR was applied to train an XGBoost model based on these features using a training set comprising 70% of the samples and with default settings. The model’s performance was evaluated using the testing set comprising 30% of the samples, yielding a ROC AUC of 1.00. We then applied the model to an independent GBM dataset by the Clinical Proteomic Tumor Analysis Consortium (CPTAC) [17]. Both datasets were normalized using the robust *Z*-score, yet we did not apply data harmonization. We assessed the classification performance of the Werner model on the 92 CPTAC samples based on the predicted class probabilities. Eight samples with probabilities within a range of ± 0.10 around the classification threshold were excluded as inconclusive. With the exception of two, all remaining samples were assigned to subtypes that match the multi-omic subtypes identified by CPTAC (Fig. 6, right). This independent validation suggests that the proteomic signature learned by the model is capturing a real biological phenotype that occurs across cohorts.

## Application example 2: classification of LUAD in serum

**Fig. 7:**
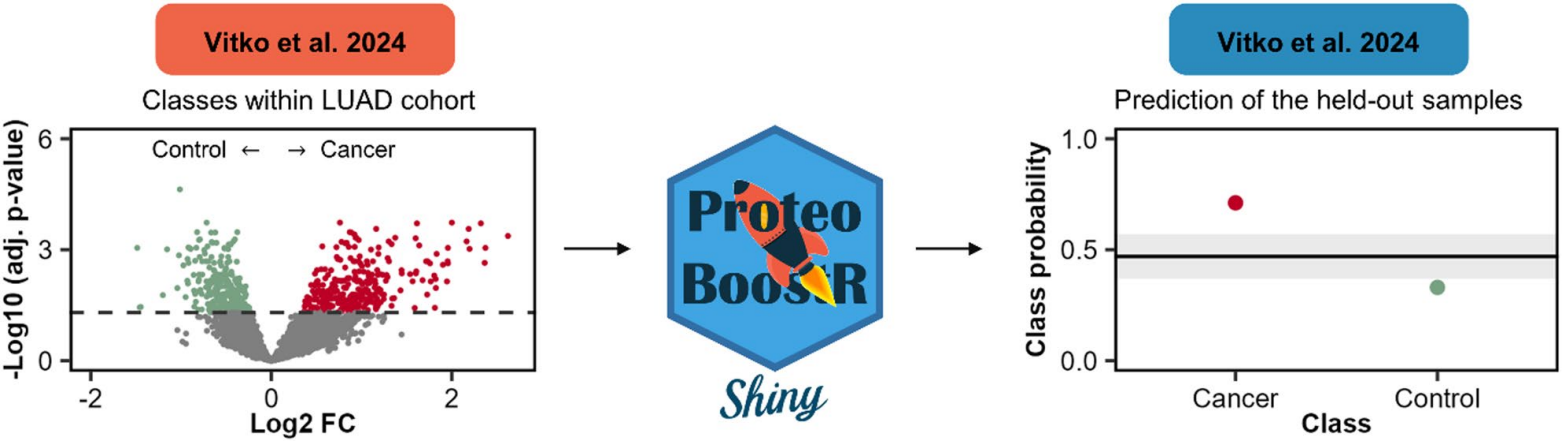
Classification of LUAD in serum using ProteoBoostR. Volcano plot of the differential abundance analysis between LUAD and control serum samples in the Vitko dataset (left). Predicted class probabilities for the held-out serum samples obtained from the trained model (right). The grey band marks the decision threshold region (default 0.1) and samples are depicted in red (cancer) and green (control). FC, fold change

To illustrate the application of ProteoBoostR in a clinically oriented setting, we used a serum proteomics dataset by Vitko et al., comprising 40 samples of patients with LUAD and non-cancer controls [18]. Two samples were set aside for hold-out evaluation. With the remaining samples, we performed a differential abundance analysis between cancer and control samples to identify proteins with strong disease-associated changes in serum (Fig. 7, left). Both the 10 most upregulated and 10 most downregulated proteins in the cancer samples with the highest absolute fold changes were selected as features for model training and ProteoBoostR was used to train a model using default settings. Finally, the trained model was applied to two held-out serum samples, yielding well-separated predicted class probabilities for cancer versus control (Fig. 7, right), consistent with the true clinical labels.

## Discussion and conclusions

We present ProteoBoostR, a comprehensive tool that brings advanced ML methods to proteomics researchers through an interactive and user-friendly platform. Complex patterns in protein abundance data can reveal important biological and clinical insights that might be missed by conventional analyses. Such patterns can uncover subtle co-regulation within protein networks, identify molecular subtypes associated with patient prognosis, or highlight pathway alterations linked to therapy response. These interdependencies shape the structure of high-dimensional proteomic data and often lead to multicollinearity and nonlinear relationships that pose challenges for simpler statistical methods such as linear regression or univariate testing, yet can be captured by ML models. In addition, missingness is a common characteristic of proteomic datasets (Fig. S1 and S2), which requires strategies capable of handling missing values. By wrapping the powerful XGBoost algorithm in a Shiny app, we enable users to create high-performance classification models without the need for coding themselves. This lowers the entry barrier for a wider range of researchers to harness ML for data analysis, making the use of these techniques easily accessible to the proteomics community.

ProteoBoostR is currently limited to binary classification, but we plan to expand it to enable multi-class classification. Further, feature selection is not yet included, as there are numerous strategies that greatly depend on the application case. Ongoing development is directed towards implementing a choice of feature selection approaches.

The application examples on proteomic subtype classification in GBM and detection of LUAD demonstrate that ProteoBoostR can both reproduce established findings and generalize predictive models to independent cohorts. As such, the tool has broad potential for clinical applications. Possible use cases include, e.g., the diagnostic classification of cancers of unknown primary and the stratification of disease subtypes that inform therapeutic decisions and patient enrollment in targeted treatment trials. Thus, ProteoBoostR may enhance the use of proteomic data in clinical trials and therapy management, thereby supporting the advancement of personalized medicine.

## Supplementary Information

Supplementary Material 1.

## Data Availability

The datasets analyzed during this study are deposited in the GitHub repository and available at https://github.com/SchillingLabProteomics/ProteoBoostR.

## Abbreviations

AUC: Area under the curve
CPTAC: Clinical Proteomic Tumor Analysis Consortium
GBM: Glioblastoma multiforme
LUAD: Lung adenocarcinoma
ML: Machine learning
MS: Mass spectrometry
ROC: Receiver operating characteristic
