# Supplementary material for: ProteoBoostR: an interactive framework for supervised machine learning in clinical proteomics
Source: Clin Proteomics. 2026 Jan 24;23:4. doi: 10.1186/s12014-026-09582-8 (PMC12849323; doi:10.1186/s12014-026-09582-8)
Supplement: Supplementary file 1 — Supplementary Material 1. [file 12014_2026_9582_MOESM1_ESM.docx]

**Supplementary Information**


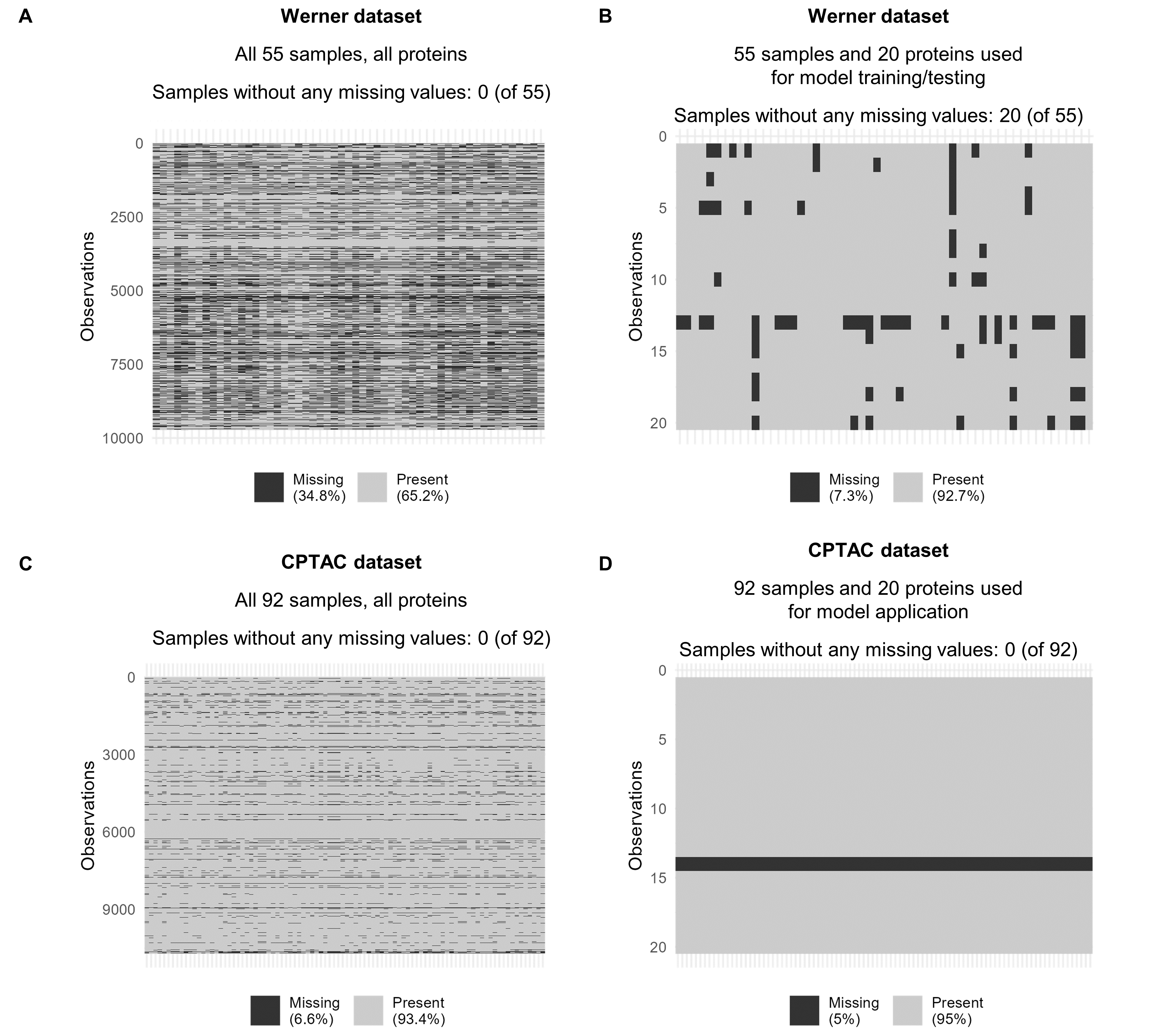


**Fig. S1 Missingness in the GBM datasets.** (A) Werner dataset – Missingness for all proteins across all 55 samples. (B) Werner dataset – Missingness for the 20 proteins used as features across all 55 samples. (C) CPTAC dataset – Missingness for all proteins across all 92 samples. (D) CPTAC dataset – Missingness for the 20 proteins used as features across all 92 samples.


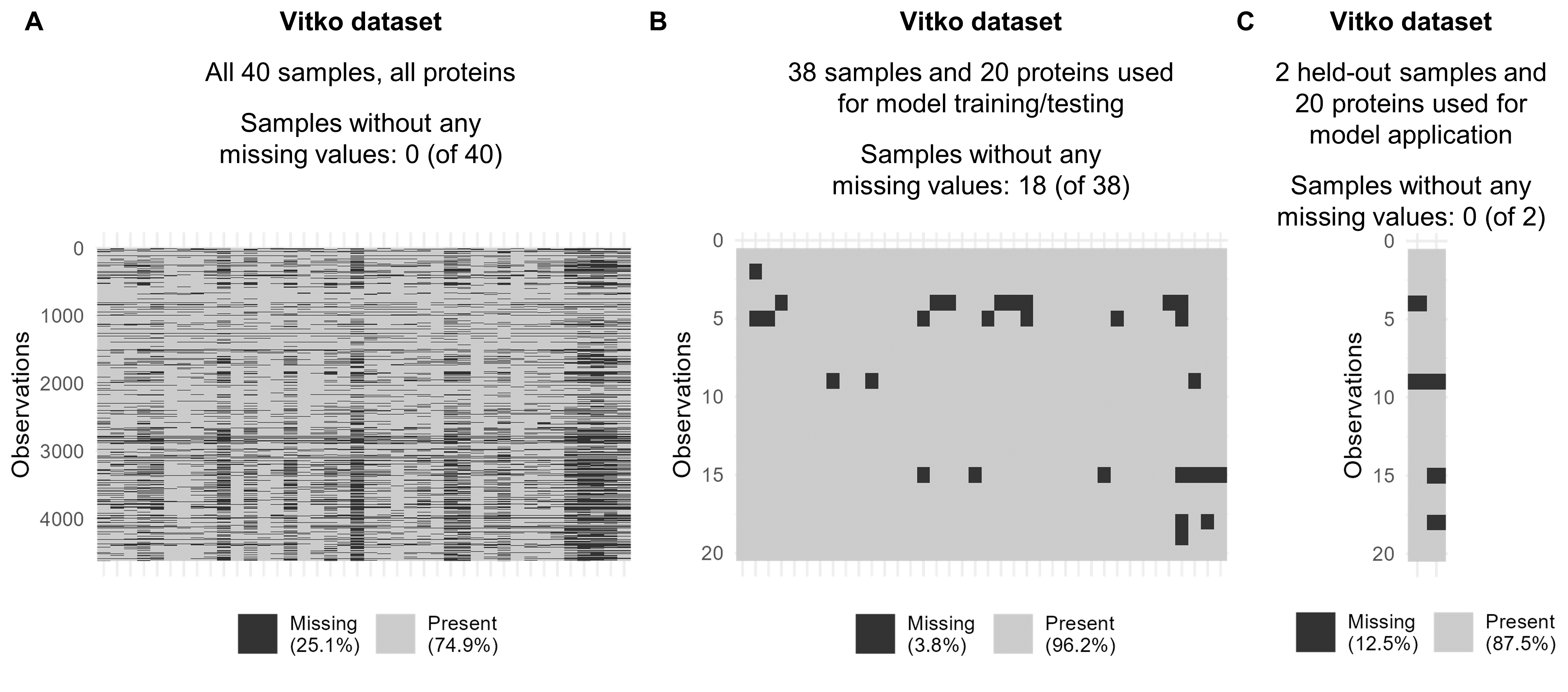


**Fig. S2 Missingness in the LUAD dataset.** (A) Vitko dataset – Missingness for all proteins across all 40 samples. (B) Vitko dataset – Missingness for the 20 proteins used as features across the 38 samples used for model training and testing. (C) Vitko dataset – Missingness for the 20 proteins used as features across the 2 held-out samples used for model application.
